# Dual-action platelet-rich fibrin in periodontal therapy: Assessing the efficacy of injectable versus metronidazole-enhanced platelet-rich fibrin in non-surgical periodontal treatment – A clinical study

**DOI:** 10.1016/j.jobcr.2025.07.028

**Published:** 2025-08-07

**Authors:** Devadharshini Chandrasekar, Burnice Nalina Kumari Chellathurai, Jaideep Mahendra, Vijayalakshmi Rajaram

**Affiliations:** Department of Periodontics, Meenakshi Ammal Dental College and Hospital, Meenakshi Academy of Higher Education and Research, Chennai, Tamil Nadu, India

**Keywords:** Periodontitis, Injectable PRF, Metronidazole-infused PRF gel, Non-surgical periodontal therapy, Adjunctive therapy

## Abstract

**Background:**

Adjunctive therapies play a crucial role in enhancing the efficacy of non-surgical periodontal therapy (NSPT) by addressing the multifactorial nature of periodontal disease. Injectable platelet-rich fibrin (i-PRF) and metronidazole-infused PRF gel have emerged as potential biomaterials that promote periodontal regeneration and antibacterial effects, respectively.

**Objective:**

To evaluate and compare the clinical efficacy of i-PRF and metronidazole-infused PRF gel as adjuncts to NSPT in patients with periodontitis.

**Method:**

ology: A randomized controlled trial was conducted on 20 periodontal sites in patients with Stage II-III periodontitis. Sites were divided into two groups (n = 10 each), receiving either i-PRF or metronidazole-infused PRF gel following NSPT. Clinical parameters, including Oral Hygiene Index (OHI), Gingival Index (GI), Bleeding on Probing (BOP), Probing Pocket Depth (PPD), and Clinical Attachment Level (CAL), were assessed at baseline, 4 weeks, and 3 months. Statistical analyses included the Mann-Whitney *U* test for intergroup comparisons and the Friedman test for intragroup comparisons.

**Results:**

Both groups showed significant improvement in PPD and CAL over the study period (p < 0.001). The metronidazole-infused PRF gel group demonstrated a more pronounced reduction in GI and BOP compared to the i-PRF group (p = 0.01 and p = 0.66, respectively). Improvements in OHI were observed in both groups but were not statistically significant.

**Conclusion:**

The study highlights the potential of metronidazole-infused PRF gel as a superior adjunct to NSPT due to its enhanced antimicrobial effects and periodontal tissue healing properties. Personalized therapeutic strategies incorporating bioactive materials can optimize periodontal treatment outcomes.

## 1 Introduction

Periodontal diseases, characterized by the progressive destruction of the supporting structures of teeth, pose a significant public health challenge with implications for both oral and systemic health.^1^ Non-Surgical Periodontal Therapy (NSPT) remains the gold standard in periodontitis management, focusing on infection control, inflammation reduction, and periodontal stabilization.^2^ However, adjunctive therapeutic strategies are increasingly being explored to enhance NSPT outcomes and optimize periodontal regeneration.

Local drug delivery (LDD) systems have gained prominence in periodontal therapy due to their ability to deliver antimicrobial agents directly to the diseased site, thereby increasing drug efficacy while minimizing systemic exposure.^3^ Among the various LDD approaches, Platelet-Rich Fibrin (PRF) has emerged as a promising autologous biomaterial owing to its regenerative and antimicrobial properties. PRF, an advanced platelet concentrate, contains a high concentration of growth factors such as platelet-derived growth factor (PDGF), transforming growth factor-beta (TGF-β), vascular endothelial growth factor (VEGF), epidermal growth factor (EGF), and insulin-like growth factor-1 (IGF-1), all of which contribute to periodontal tissue repair and regeneration.^4^ Additionally, PRF provides a three-dimensional fibrin matrix that supports cellular migration, proliferation, and differentiation, further enhancing its regenerative potential.^5^

A more recent innovation in platelet-based therapeutics is liquid-injectable PRF (i-PRF), introduced by Miron et al. Unlike conventional PRF, i-PRF remains in a liquid state for a short duration before forming a fibrin gel, allowing for enhanced cellular recruitment and sustained growth factor release over an extended period.^6^ The incorporation of i-PRF into periodontal therapy has demonstrated significant improvements in fibroblast and osteoblast activity, type I collagen gene expression and periodontal ligament cell proliferation.^7^

In the present study, we propose an innovative approach that combines the regenerative potential of PRF with the antimicrobial efficacy of metronidazole to develop a metronidazole-infused PRF gel as a novel local drug delivery system. Metronidazole, an established antibiotic with high specificity against anaerobic bacteria implicated in periodontitis, offers a targeted antibacterial effect.^8^ When integrated into PRF, this dual-action biomaterial is hypothesized to provide both antimicrobial activity and regenerative support, enhancing clinical outcomes in periodontitis treatment.

Therefore, the aim of the present study was to evaluate and compare the clinical efficacy of Injectable Platelet-Rich Fibrin (i-PRF) and Metronidazole-Infused PRF Gel as adjuncts to Non-Surgical Periodontal Therapy.

## 2 Materials and methods

### 2.1 Study design and ethical approval

**Fig. 1:**
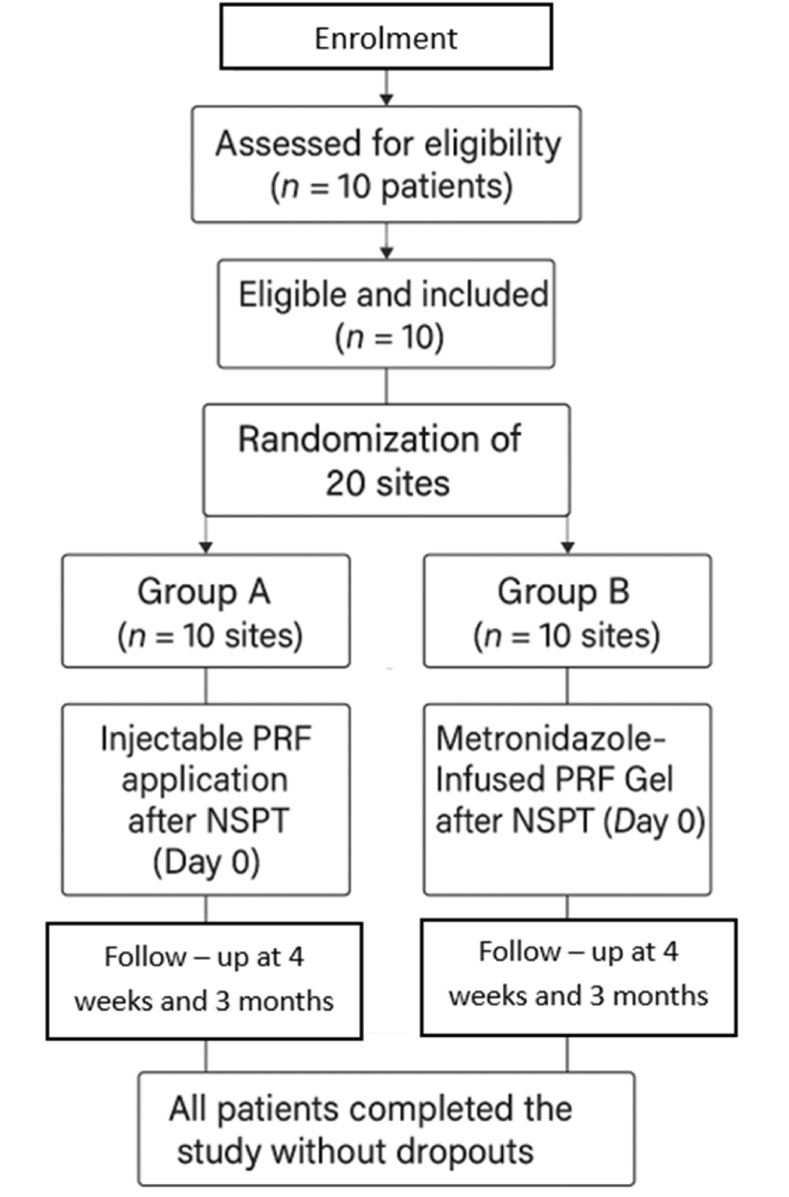
– Study design flowchart.

A randomized controlled clinical trial was conducted in the Department of Periodontology, Meenakshi Ammal Dental College and Hospital, Meenakshi Academy of Higher Education & Research (MAHER), Chennai. Ethical clearance for the study was obtained from the Institutional Ethical Committee prior to the commencement of the trial (MADC/IEC/I/06/2024) (Fig. 1). The study adhered to the principles outlined in the Declaration of Helsinki. Written informed consent was obtained from all participants prior to enrolment.

### 2.2 Blinding protocol

To minimize bias, a single-blind study design was employed. The investigator responsible for clinical measurements and outcome assessments was blinded to the group allocation of the treatment sites. Group allocation (i.e., whether the site received Injectable Platelet-Rich Fibrin or Metronidazole-infused PRF gel) was concealed during all follow-up assessments. The operator performing the treatment procedures was not blinded due to the nature of material preparation and administration, but had no involvement in clinical evaluations or data analysis. This blinding approach ensured objective and unbiased measurement of clinical outcomes.

### 2.3 Study population and sample size

A total of 10 systemically healthy patients, both males and females, aged above 18 years and diagnosed with Stage II to III, Grade A or B periodontitis (as per 2017 Classification of Periodontal Diseases), were recruited.^9^ Each participant presented with a minimum of two periodontal sites per quadrant exhibiting probing depth ≥5 mm following Phase I therapy. A total of 20 periodontal sites were included and randomly allocated into two groups (n = 10 sites per group) using the coin toss method: (Figure – 1)

• **Group A**: Received subgingival Injectable Platelet-Rich Fibrin (i-PRF) following non-surgical periodontal therapy (NSPT).
• **Group B**: Received subgingival Metronidazole-Infused PRF Gel following NSPT.

Participants included in the study were systemically healthy individuals aged 18 years and above. They were diagnosed with Stage II to III periodontitis and classified as Grade A or B based on the 2017 Classification of Periodontal Diseases.^9^ Eligible participants presented with probing pocket depths of ≥5 mm on at least two teeth in each quadrant following Phase I therapy. Only individuals who demonstrated good oral hygiene compliance during the initial evaluation were considered for enrolment. Subjects were excluded if the selected teeth exhibited structural defects such as caries, deep abrasion, demineralization, extensive restorations, or any signs of pulpal involvement. Additionally, those with a history of periodontal surgery in the affected sites within the past year, or those currently on medications known to interfere with wound healing, were not considered. Pregnant and lactating women, smokers, alcoholics, and individuals with systemic conditions that could compromise periodontal healing were excluded. Teeth with Grade II or III furcation involvement and patients presenting with Stage IV, Grade C periodontitis were also omitted from the study. The demographic details of the study participants are summarized in Table 1.

**Table 1:** Demographic characteristics of the study participants (n = 10).

Variable	Value/Distribution
Age (Mean ± SD)	35.6 ± 7.2 years
Age Range (years)	24–48
Gender (n)	Male: 6, Female: 4
Stage of Periodontitis (n)	Stage II: 4, Stage III: 6
Grade of Periodontitis (n)	Grade A: 3, Grade B: 7
Sites Allocated to Group A (i-PRF)	10 periodontal sites
Sites Allocated to Group B (Metronidazole-PRF)	10 periodontal sites
Systemic Status	All participants systemically healthy
Smoking Status	Non-smokers

### 2.4 Clinical parameters

Clinical parameters including the Oral Hygiene Index–Simplified (OHI-S), Gingival Index (GI), Bleeding on Probing (BOP), Probing Pocket Depth (PPD), and Clinical Attachment Level (CAL) were recorded at baseline (Day 0), 4 weeks, and 3 months. To ensure reproducible probe placement, a customized acrylic stent was fabricated for each patient, with the lower border serving as a fixed reference point. PPD and CAL measurements were made through the stent using a Williams periodontal probe at each time point, thereby minimizing examiner variability and enhancing accuracy. OHI-S and GI scores were assessed according to Greene & Vermillion and Loe & Silness respectively, while BOP was recorded dichotomously (present/absent) at six sites per tooth.^10^^,^^11^ All measurements were performed by a single calibrated examiner to further standardize data collection across visits.

### 2.5 Pre-treatment procedure

#### 2.5.1 Injectable PRF (i-PRF) – Mourao protocol (2010)^6^

A volume of 20 mL of venous blood was drawn from the antecubital vein using sterile plastic tubes without anticoagulant. Blood samples were immediately centrifuged at 700 rpm for 3 min using a standard tabletop centrifuge. Post-centrifugation, the upper yellow-orange layer (i-PRF) was carefully aspirated into sterile insulin syringes (approximately 1 mL per tube) for immediate subgingival application.

### 2.6 Metronidazole-infused PRF gel

A volume of 10 mL of venous blood was collected and transferred into sterile glass tubes without anticoagulant and centrifuged at 2700 rpm for 12 min. The resultant PRF clot (second layer) was collected and transferred to an incubated test tube containing 2 mL of 0.5 % Metronidazole (1/20 of a 200 mg metronidazole tablet). To facilitate gelation, PRF was allowed to incubate for 10 min at room temperature until a gel-like consistency was achieved (Figure - 2).

**Fig. 2:**
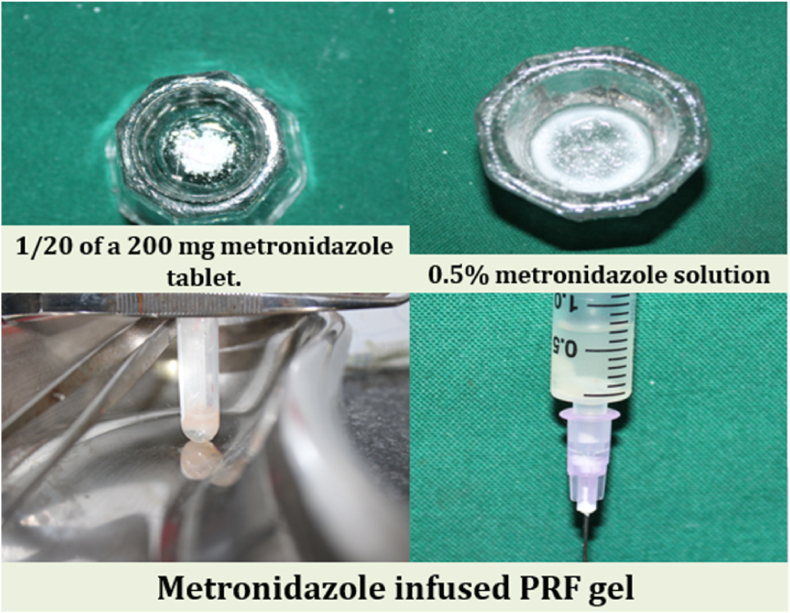
Metronidazole infused PRF Gel.

### 2.7 Intellectual property and patent disclosure

The Metronidazole-infused injectable Platelet-Rich Fibrin (i-PRF) gel utilized in this study is a novel, patented formulation designed to provide both regenerative and antimicrobial benefits in periodontal therapy. The method for its preparation has been documented and published as an official patent under Indian Patent Application No. 202441071478 A, titled *“A Process of Preparation of Metronidazole Infused i-PRF Gel for the Management of Periodontal Pocket.”* This application was published in The Patent Office Journal No. 40/2024, dated October 4, 2024. This intellectual property is protected under Indian patent law, and all associated rights are retained by the inventors. The formulation was developed exclusively for research purposes and used under appropriate ethical and institutional approvals within the scope of this clinical study.

### 2.8 Non-surgical periodontal therapy (NSPT)

All patients received Phase I therapy (scaling and root planing) under local anaesthesia using Gracey curettes. Clinical parameters were recorded immediately after therapy and considered as baseline (Day 0).

• In **Group A**, i-PRF was administered sub gingivally into the selected periodontal pockets (Figure - 3).

**Fig. 3:**
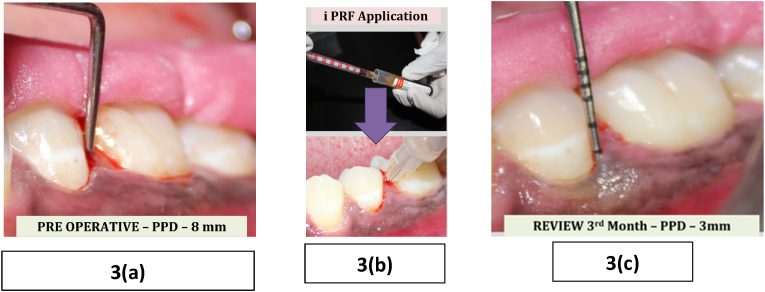
– Group A – Subgingival application of i-PRF and follow up (3a) Baseline clinical photograph showing a probing pocket depth (PPD) of 8 mm prior to intervention. (3b) Subgingival administration of injectable PRF (i-PRF) following non-surgical periodontal therapy (NSPT) using a blunt-tipped cannula. (3c) Post-operative clinical photograph at 3-month review visit showing significant reduction in probing depth to 3 mm.
• In **Group B**, Metronidazole-Infused PRF Gel was placed into the respective sites using a sterile syringe (Figure - 4).

**Fig. 4:**
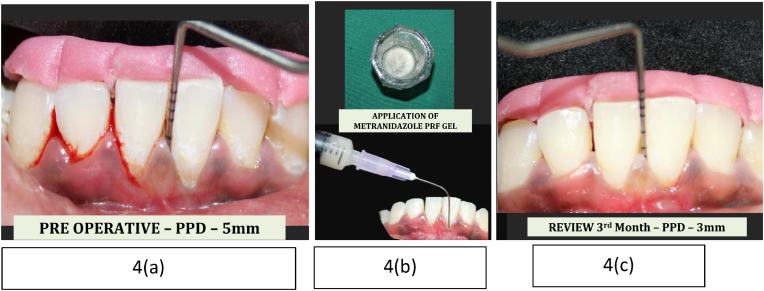
Group B Subgingival application of Metronidazole infused PRF gel and follow - up (4a) Baseline clinical photograph showing a probing pocket depth (PPD) of 5 mm prior to treatment. (4b) Preparation and subgingival delivery of Metronidazole-infused PRF gel using a syringe following non-surgical periodontal therapy (NSPT). (4c) Post-operative clinical photograph at 3-month review visit demonstrating reduction in probing depth to 3 mm.

### 2.9 Post-treatment and follow-up

Patients were given standard post-treatment instructions and were monitored for any adverse reactions. Clinical re-evaluation was performed at 1 month (4 weeks) and 3 months post-treatment. The primary outcome measures were reduction in Probing Pocket Depth (PPD) and gain in Clinical Attachment Level (CAL). Secondary outcome measures included improvements in GI, BOP, and OHI-S.

### 2.10 Statistical analysis

Statistical analysis was performed using SPSS software version 22.0 (IBM Corp., Armonk, NY, USA). Mean and standard deviation were calculated for each clinical parameter. Intergroup and intragroup comparisons were made using paired and unpaired t-tests, respectively. A p-value <0.05 was considered statistically significant.

## 3 Results

Continuous variables were expressed as Median ± Inter Quartile Range (IQR), while categorical variables were presented as frequency (n) and percentage (%). The data were subjected to the Shapiro-Wilk test to assess normality. As the data did not follow a normal distribution, non-parametric tests were employed for analysis. Mann–Whitney *U* test was used for intergroup comparisons, and Friedman test was applied for intra-group comparisons. A p-value of <0.05 was considered statistically significant.

The Oral Hygiene Index (OHI) scores showed a transient increase at the 4-week interval compared to baseline in both the SRP ± i-PRF and SRP ± Metronidazole PRF gel groups, followed by a reduction at the 3-month follow-up. These fluctuations, however, were not statistically significant within or between the groups (Table 2). Gingival Index (GI) scores in the SRP ± Metronidazole PRF gel group demonstrated a notable decrease from 2.0 ± 1.0 at baseline to 1.0 ± 0.0 at 4 weeks, indicating early resolution of gingival inflammation. In contrast, no improvement was observed in the SRP ± i-PRF group at the 4-week mark. By the end of 3 months, both groups exhibited a significant reduction in GI scores to 1.0 ± 0.0 compared to baseline values, and these changes were statistically significant (p < 0.05). (Table 1). Probing Pocket Depth (PPD) significantly reduced in both groups over the course of the study. In the SRP ± i-PRF group, the median PPD decreased from 5 ± 0.0 mm at baseline to 3 ± 0.0 mm at both 4 weeks and 3 months. A similar trend was observed in the SRP ± Metronidazole PRF gel group, where the median PPD dropped from 6 ± 2.0 mm at baseline to 3 ± 0.0 mm at both subsequent time points. These reductions were statistically significant in both groups (p < 0.001) (Table 3). Clinical Attachment Loss (CAL) also showed a statistically significant reduction across the study duration. In the SRP ± i-PRF group, CAL values declined from 5 ± 2.75 mm at baseline to 3 ± 2.0 mm at 4 weeks and remained stable at 3 ± 2.0 mm at 3 months. The SRP ± Metronidazole PRF gel group exhibited an even greater reduction, with CAL values decreasing from 6 ± 3.5 mm at baseline to 3 ± 1.5 mm at 4 weeks, and further to 3 ± 0.0 mm by the 3-month mark. These reductions were statistically significant in both groups (p < 0.001) (Table 3). Bleeding on probing (BOP) showed a marked reduction in the number of participants with positive findings across both groups. In the SRP ± i-PRF group, BOP was observed in 7 participants (58.3 %) at baseline, decreasing to 5 participants (41.7 %) at 3 months. Similarly, in the SRP ± Metronidazole PRF gel group, the number reduced from 8 participants (66.7 %) at baseline to 4 participants (33.3 %) at the end of the study, reflecting improved gingival health (Table 4).

**Table 2:** Intra group and Intergroup comparisons of Oral Hygiene Index and Gingival Index between two study groups at various time points.

		SRP ± IPRF Gel (Median ± IQR)	SRP ± Metronidazole PRF Gel (Median ± IQR)	p-value^b^
Oral Hygiene Index (OHI)	Baseline	1.2 ± 0.3	1.2 ± 0.8	0.67
	4 weeks	1.5 ± 0.95	1.5 ± 0.5	0.47
	3 months	1.2 ± 0.5	1.35 ± 0.5	0.71
	**p-value**^a^	0.27	0.14	–
Gingival Index (GI)	Baseline	2.0 ± 1.0	2.0 ± 1.0	0.37
	4 weeks	2.0 ± 1.0	1.0 ± 0.0	0.08
	3 months	1.0 ± 0.0	1.0 ± 0.0	0.51
	**p-value**^a^	**0.02∗**	**0.01∗**	–

∗p-value <0.05 – statistically significant.

a Intragroup comparisons (Friedman test).

b Intergroup comparisons (Man Whitney test).

**Table 3:** Intra group and Intergroup comparisons of Probing pocket depth and Clinical attachment loss between two study groups at various time points.

		SRP ± IPRF Gel (Median ± IQR)	SRP ± Metronidazole PRF Gel (Median ± IQR)	p-value^b^
Probing pocket depth	Baseline	5.0 ± 0.0	6 ± 2.0	1.00
	4 weeks	3.0 ± 0.0	3 ± 0.0	0.16
	3 months	3.0 ± 0.0	3 ± 0.0	0.44
	**p-value**^a^	**<0.001∗**	**<0.001∗**	–
Clinical attachment loss	Baseline	5 ± 2.75	6 ± 3.5	0.67
	4 weeks	3.0 ± 2.0	3 ± 1.5	0.41
	3 months	3.0 ± 2.0	3 ± 0.0	0.39
	**p-value**^a^	**<0.001∗**	**<0.001∗**	–

∗p-value <0.05 – statistically significant.

a Intragroup comparisons (Friedman test).

b Intergroup comparisons (Man Whitney test).

**Table 4:** Intra group and Intergroup comparisons of Bleeding on probing between two study groups at various time points.

		SRP ± IPRF Gel N (%)	SRP ± Metronidazole PRF Gel N (%)	p-value^b^
**Presence of Bleeding on probing**	Baseline	7 (58.3)	8 (66.7)	0.22
	4 weeks	4 (33.3)	4 (33.3)	0.12
	3 months	5 (41.7)	4 (33.3)	0.12
	**p-value**^a^	0.5	0.66	0.55

∗p-value <0.05 – statistically significant.

a Intragroup comparisons (Fried man test).

b Intergroup comparisons (Chi square test).

## 4 Discussion

The present study was designed to evaluate and compare the clinical efficacy of Non-Surgical Periodontal Therapy (NSPT) with adjunctive use of injectable Platelet-Rich Fibrin (i-PRF) and SRP with Metronidazole-infused PRF gel, a novel drug-delivery formulation developed and patented by the authors. With the growing emphasis on minimally invasive and biologically driven regenerative therapies, there is a pressing need to develop bioactive local delivery systems that not only support tissue healing but also possess antimicrobial properties. This study aimed to bridge that gap by evaluating a dual-action therapeutic approach combining the regenerative potential of PRF with the antimicrobial efficacy of metronidazole.

In the present investigation, the Oral Hygiene Index (OHI) scores showed a slight increase at the 4-week interval from baseline in both groups, followed by a reduction at the 3-month follow-up, although these changes were not statistically significant. The temporary rise in OHI scores may be attributed to transitional oral hygiene behaviours or plaque accumulation during initial healing. Similar trends were observed in the study by Jonsson & Ohrn, where early fluctuations in OHI scores stabilized over time following non-surgical periodontal therapy.^12^ Medaiah et al. also reported non-significant changes in OHI following SRP in both control and adjunctive therapy groups, highlighting that OHI values may not directly reflect clinical periodontal status during short-term follow-ups.^13^

The Gingival Index (GI) scores in the SRP ± Metronidazole PRF gel group significantly reduced from 2.0 ± 1.0 at baseline to 1.0 ± 0.0 at 4 weeks, demonstrating early resolution of gingival inflammation. In contrast, the SRP ± i-PRF group did not show improvement at 4 weeks but achieved a significant reduction to 1.0 ± 0.0 by 3 months. This delayed improvement may be attributed to the absence of a targeted antimicrobial agent in i-PRF alone. The findings are consistent with Figuero et al. in their systematic review and meta analysis reported enhanced gingival healing with adjunctive antimicrobial agents.^14^ Similarly, Mehravani et al. found that metronidazole-based local delivery systems significantly reduced gingival inflammation in chronic periodontitis patients, supporting the present study's findings.^15^ However, Rakhewar et al. suggested that the regenerative effect of i-PRF alone may take longer to manifest in clinical indices such as GI, indicating a delayed yet stable improvement in gingival health.^16^

The Probing Pocket Depth (PPD) showed a statistically significant reduction in both groups. In the SRP ± i-PRF group, PPD decreased from 5 ± 0.0 mm to 3 ± 0.0 mm, and in the SRP ± Metronidazole PRF gel group from 6 ± 2.0 mm to 3 ± 0.0 mm at the 3-month interval. These results underscore the effectiveness of adjunctive PRF therapies in improving pocket depth. Miron et al. demonstrated a comparable reduction in PPD using PRF-based biomaterials in chronic periodontitis patients.^17^ Furthermore, Soysa et al. observed significant pocket depth reductions when combining SRP with local antibiotic-infused gels, corroborating the enhanced clinical benefit observed in the Metronidazole PRF group.^18^ In contrast, Van der Weijden & Timmerman reported only modest improvements with SRP alone, reaffirming the adjunctive advantage seen in the current trial.^19^

The Clinical Attachment Loss (CAL) also demonstrated marked improvements. The SRP ± i-PRF group showed a reduction from 5 ± 2.75 mm to 3 ± 2.0 mm, while the SRP ± Metronidazole PRF gel group improved from 6 ± 3.5 mm to 3 ± 0.0 mm over the 3-month period. These findings indicate substantial periodontal tissue regeneration, particularly in the Metronidazole PRF group. Miron et al. emphasized the potential of PRF in enhancing attachment levels due to its sustained release of growth factors.^17^ Likewise, Omar et al. reported that antibiotic-augmented platelet concentrates demonstrated superior CAL gains compared to conventional PRF, aligning closely with the present results.^20^ Contrarily, Varshney et al. found inconsistent CAL improvements with autologous platelet products, suggesting variability in outcomes based on PRF preparation and adjunctive agents.^21^

Bleeding on Probing (BOP) showed a favourable decline in both groups. In the SRP ± i-PRF group, BOP decreased from 58.3 % to 41.7 %, while in the Metronidazole PRF group, it reduced from 66.7 % to 33.3 % at the 3-month review. The reduction in gingival bleeding reflects the anti-inflammatory and antimicrobial efficacy of the interventions. These outcomes are in line with Hammami & Nasri, who observed a significant reduction in BOP with antibiotic-containing gels in chronic periodontitis.^22^ Moreover, Vuckovic et al. reported similar bleeding control when PRF was combined with adjunctive anti-infective therapy, reinforcing the observations made in this study.^23^

The principal strength and novelty of this study lie in the formulation, patenting, and clinical validation of a Metronidazole-infused injectable Platelet-Rich Fibrin (i-PRF) gel—a dual-functional therapeutic biomaterial designed to address both microbial load and tissue regeneration. Unlike conventional local drug delivery systems that primarily offer antimicrobial action without regenerative potential, or traditional platelet concentrates that provide growth factors but lack targeted antimicrobial properties, this innovative formulation synergistically combines regenerative and antimicrobial capabilities in a single injectable system. The patented technology not only enhances site-specific delivery but also extends the bioactivity window of both components, ensuring sustained therapeutic effects. Its integration into non-surgical periodontal therapy (NSPT) represents a paradigm shift in managing periodontal pockets, especially in patients with a high microbial burden, where both infection control and soft tissue healing are critical. This advancement could significantly broaden the clinical scope of minimally invasive periodontal therapies.

However, the present study has several limitations. The relatively small sample size and short duration of follow-up restrict the generalizability and long-term interpretation of the outcomes. Patient compliance and oral hygiene maintenance during the study period could have also influenced the results. In addition, histological analysis and microbial quantification were not included, which could have provided deeper mechanistic insights.

Future studies should focus on long-term multicentre clinical trials with larger sample sizes to validate the clinical consistency and safety of the Metronidazole-infused i-PRF gel. Moreover, further exploration of its use in advanced periodontal lesions, peri-implant diseases, and its histomorphometric effects will help in expanding the clinical application of this novel formulation. Investigation into other combinations of growth factors and antibiotics in PRF matrices could also open new frontiers in periodontal regenerative therapy.

## 5 Conclusion

Within the constraints of this randomized controlled trial, adjunctive use of both i-PRF and the novel Metronidazole-infused PRF gel yielded significant improvements in probing pocket depth, clinical attachment level, and bleeding on probing, while oral hygiene index fluctuations remained minimal, demonstrating enhanced periodontal healing when combined with SRP. Crucially, the Metronidazole-infused PRF gel is a first-in-class, patented autologous biomaterial that uniquely integrates sustained growth factor release with targeted antimicrobial action, offering a powerful dual-function therapeutic strategy. This offers a transformative approach to non-surgical periodontal therapy and establishes a new benchmark for integrated regenerative and antimicrobial periodontal care.

## Institutional Ethics Committee (IEC) approval

Approved by the Institutional Ethics Committee of Meenakshi Ammal Dental College and Hospital (Approval No.: MADC/IEC/I/06/2024).

## Author contributions

Dr. Devadharshini Chandrasekar: Conceptualization, Data Collection, Manuscript Drafting

Dr. Burnice Nalina Kumari C.: Supervision, Manuscript Review, Correspondence

Dr. Jaideep Mahendra: Methodology, Critical Revision, Final Approval

Dr. Vijayalakshmi Rajaram: Data Analysis, Critical Revision.

## Funding source

This research did not receive any specific grant from funding agencies in the public, commercial, or not-for-profit sectors.

## Sources of funding

This study did not receive any specific grant from funding agencies in the public, commercial, or not-for-profit sectors. The research was conducted using institutional resources and personal contributions by the investigators.

There are no financial interests or external sponsors influencing the outcomes of this research.

## Declaration of competing interest

The authors declare that they have no known competing financial interests or personal relationships that could have appeared to influence the work reported in this paper.
